# Clinical impact of ultra deep versus Sanger sequencing detection of minority mutations on HIV-1 drug resistance genotype interpretation after virological failure

**DOI:** 10.1186/1471-2334-14-S2-O1

**Published:** 2014-05-23

**Authors:** Sofiane Mohamed, Guillaume Penaranda, Dimitri Gonzalez, Claire Camus, Hacène Khiri, Ronan Boulmé, Chalom Sayada, Patrick Philibert, Daniel Olive, Philippe Halfon

**Affiliations:** 1 Alphabio Laboratory, Marseille, France; 2 Advanced Biological Laboratories (ABL), Luxembourg, Luxembourg; 3 European Hospital, Marseille, France; 4 Paoli Calmettes Institute, Tumor Immunology Laboratory and INSERM Center of Cancer Research, Marseille, France

## Aim

Drug resistance mutations are routinely detected using standard Sanger sequencing, which does not detect minor variants with a frequency below 20%. The impact of detecting minor variants generated by ultra-deep sequencing (UDS) on HIV drug-resistance (DR) interpretations has not yet been studied.

## Methods

Fifty HIV-1 patients who experienced virological failure were included in this retrospective study. The HIV-1 UDS protocol was performed using the GS Junior (Roche 454 Life Sciences Branford, CT). This UDS protocol allowed the detection and quantification of minor and major HIV-1 protease and reverse transcriptase variants related to genotypes A, B, C, E, F and G. DeepChek®-HIV (ABL, SA and TherapyEdgeTM, USA) simplified drug resistance (DR) interpretation software was used to compare Sanger sequencing and UDS at two different thresholds (≥1% and ≥20%). DeepChek®-HIV utilizes the ANRS, HIVdb and Rega algorithms.

## Results

The total time required for the UDS protocol was found to be approximately three times longer than Sanger sequencing with equivalent reagent costs. UDS detected all of the mutations found by population sequencing and identified additional resistance variants in all patients, primarily by using 1% sensitivity. An analysis of DR revealed a total of 643 and 224 clinically relevant mutations by UDS and Sanger sequencing, respectively. Three resistance mutations with >20% prevalence were detected solely by UDS: A98S (23%), E138A (21%) and V179I (25%). A significant difference in the DR interpretations for 19 antiretroviral drugs was observed between the UDS and Sanger sequencing methods. Y181C and T215Y were the most frequent mutations associated with interpretation differences. The major discrepancies between Sanger and UDS were primarily found at the 1% threshold in the three algorithms.

## Conclusion

UDS was more sensitive than the standard Sanger sequencing. A combination of UDS and DeepChek® software for the interpretation of DR results saved a considerable amount of time and would help clinicians provide suitable treatments. A cut-off of 1% allowed a better characterization of the viral population by identifying additional resistance mutations and improving the DR interpretation.

